# Measurement Method for Nonlinearity in Heterodyne Laser Interferometers Based on Double-Channel Quadrature Demodulation

**DOI:** 10.3390/s18092768

**Published:** 2018-08-22

**Authors:** Haijin Fu, Ruidong Ji, Pengcheng Hu, Yue Wang, Guolong Wu, Jiubin Tan

**Affiliations:** Institute of Ultra-Precision Optoelectronic Instrument Engineering, Harbin Institute of Technology, Harbin 150001, China; haijinfu@hit.edu.cn (H.F.); safeup@163.com (R.J.); wy206@stu.hit.edu.cn (Y.W.); garywu@hit.edu.cn (G.W.); jbtan@hit.edu.cn (J.T.)

**Keywords:** laser interferometry, heterodyne laser interferometer, nonlinearity measurement, double-channel quadrature demodulation

## Abstract

The phase quadrature measurement method is capable of measuring nonlinearity in heterodyne laser interferometers with picometer accuracy whereas it cannot be applied in the new kind of heterodyne interferometers with bidirectional Doppler frequency shift especially in the condition of non-uniform motion of the target. To solve this problem, a novel measurement method of nonlinearity is proposed in this paper. By employing double-channel quadrature demodulation and substituting the external reference signal with internal ones, this method is free from the type of heterodyne laser interferometer and the motion state of the target. For phase demodulation, the phase differential algorithm is utilized to improve the computing efficiency. Experimental verification is carried out and the results indicate that the proposed measurement method achieves accuracy better than 2 pm.

## 1. Introduction

Heterodyne laser interferometers are widely applied in precision metrology, nanotechnology, and lithography due to their high accuracy and robustness [1,2,3]. With the development of science and technology, it is badly in need of laser interferometers with picometer accuracy [4] such as the next-generation laser interferometers. However, the improvement of the measurement accuracy of heterodyne laser interferometers is seriously restricted by the nonlinearity [5,6,7], i.e., the periodic nonlinear error. The measurement method of nonlinearity as an indispensable auxiliary tool plays an essential role in developing the next-generation laser interferometers.

Several methods for measuring the nonlinearity of heterodyne laser interferometers have been developed [8,9,10]. The most widely used method is the frequency domain method [8]. This method is simple and convenient to operate, but it is only applicable to the cases with constant velocity. Moreover, limited by the background noise of the spectrometer, picometer accuracy is not available for this method. Another method is the displacement comparison method [9] when compared to an identical displacement. By subtracting the result of an X-ray interferometer from that of a laser interferometer, the nonlinearity can be obtained. Picometer accuracy is easily achieved by this method while the X-ray interferometer is difficult to be replicated due to technique and cost issues and the measurement process is complex because of the special property of X-rays. Benefited from lock-in amplification, the phase quadrature measurement method [10] is promising in a nonlinearity measurement up to picometer accuracy. In this method, the reference signal of the interferometer serves as the external reference signal of a lock-in amplifier in which there is a phase-locked loop that tracks the frequency of the external reference signal. Then a pair of quadrature signals with the same frequency are generated internally for the phase demodulation. The frequency of the external reference signal is expected to keep constant or to vary slowly. Otherwise, the phase-locked loop might work in the tracing state rather than the locked state [11], which will cause an error for phase demodulation. For the traditional heterodyne laser interferometers, the frequency of the reference signal is of constant frequency, i.e., the split frequency of the laser source, so there is no such problem. For the next generation heterodyne laser interferometers [12,13], most of them adopt an optical configuration with bidirectional Doppler frequency shift (DFS), i.e., the measurement and reference signals have equal DFS but come with an opposite sign. When the target is in fast and non-uniform motion, the frequency of the reference signal will change rapidly. In this case, the existing phase quadrature measurement method is not applicable.

This paper presents a novel measurement method for nonlinearity in heterodyne laser interferometers, which adopts the architecture of double-channel quadrature demodulation with internal references and, thereby, is able to break through the limits in type of heterodyne laser interferometers and in motion state of the target. In addition, the phase differential algorithm is utilized to improve the computing efficiency. Experiments are carried out to verify the performance of the proposed method.

## 2. Measurement Method for Nonlinearity Based on Double-Channel Quadrature Demodulation

**Figure 1:**
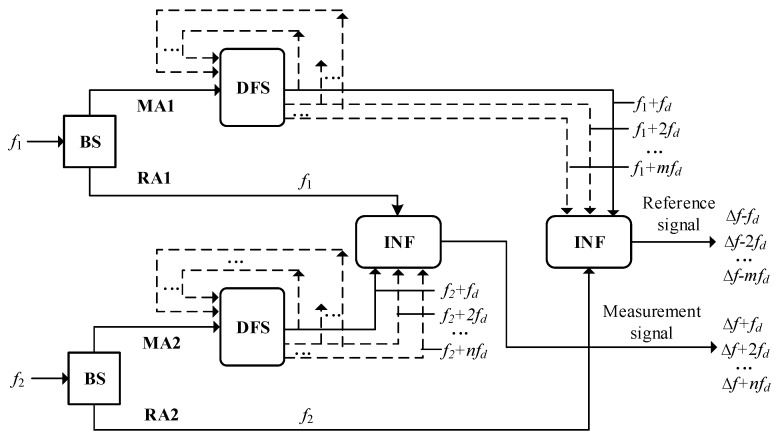
Schematic of formation mechanism of the reference and measurement signals in heterodyne laser interferometers with spatially separated optical paths and bidirectional DFS. BS: beam splitter, MA: measurement arm, RA: reference arm, DFS: Doppler frequency shift, INF: interference.

The nonlinearity in heterodyne laser interferometers originates from the frequency mixing in the reference and measurement arms [14,15,16]. In recent years, to avoid the nonlinearity, heterodyne interferometers with spatially separated optical paths have been developed [12,13]. In this kind of interferometers, the reference and measurement beams with slightly different frequencies are separated spatially before interference. In theory, there is no frequency mixing and, thereby, the nonlinearity can be completely avoided. However, an experimental study reveals that there is still nonlinearity in this kind of interferometer and its source is ascribed to the multi-order DFS induced by ghost reflection [17,18], i.e., the laser beams are repeatedly reflected between the beam splitter and the target. To improve the resolution by a factor of two, this kind of heterodyne laser interferometer usually adopts an optical layout with bidirectional DFS. Figure 1 shows the schematic of the formation mechanism of the reference and measurement signals in this type of interferometer. Considering the multi-order DFS, the reference and measurement signals can be expressed by the equation below.

(1) $$\left\{ \begin{array}{l} I_{r}=A\cos\left(\Delta \omega t-\Delta \varphi \;\right)+\alpha_{1}\cos(\Delta \omega t-2\Delta \varphi )\;+\alpha_{2}\cos(\Delta \omega t-3\Delta \varphi )+\ldots \\ \;\;\;=A\cos\left(\Delta \omega t-\Delta \varphi \right)+\sum_{m}\alpha_{m}\cos\left(\Delta \omega t\;+\;\theta_{m}\right)\\ I_{m}=B\cos\left(\Delta \omega t+\Delta \varphi \right)+\beta_{1}\cos\left(\Delta \omega t+2\Delta \varphi \right)\;\;+\beta_{2}\cos\left(\Delta \omega t+3\Delta \varphi \right)+\ldots \\ \;\;\;\;=B\cos\left(\Delta \omega t+\Delta \varphi \right)+\sum_{n}\beta_{n}\cos\left(\Delta \omega t+\delta_{n}\right), \end{array}\right.$$

where *A* and *B* are the amplitudes of the intended reference and measurement signals, respectively. $\alpha_{m}\;(m\;=\;1,\;2,\;3,\;\ldots )$ and $\beta_{n}\;(n\;=\;1,\;2,\;3,\;\ldots )$ are the amplitudes of the *m*th and *n*th order nonlinear harmonics in the reference and measurement signals, respectively, generally $\alpha_{m}\;(m\;=\;1,\;2,\;3,\;\ldots )\;\ll \;A$ and $\beta_{n}\;(n\;=\;1,\;2,\;3,\;\ldots )\;\ll \;B$. $∆\omega \;=\;2\pi ∆{f\;=\;2\pi (f}_{1}-{\;f}_{2})$ is the beat frequency, $f_{1}$ and $f_{2}$ are the optical frequencies of the dual-frequency laser source. $\theta_{m}$= −(*m* + 1)Δφ and $\delta_{n}$ = (*n* + 1)Δφ. Δφ is the measured phase and can be calculated by using Equation (2).

(2) $$\Delta \varphi \;\;=\;\;2\pi \int f_{d}\;dt,$$

where $f_{d}$ is the DFS.

**Figure 2:**
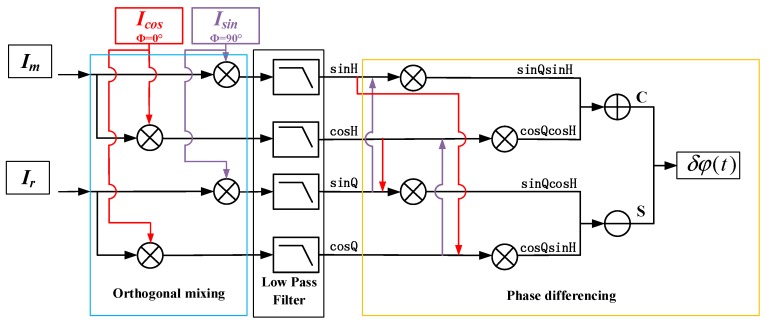
Schematic of the measurement method for nonlinearity in heterodyne laser interferometers based on a double-channel quadrature demodulation.

As indicated by Equation (1), the reference and measurement signals for the new type interferometer have equal DFS but come with an opposite sign. Therefore, the frequency of the reference signal is also determined by the motion state of the target. When the target is in non-uniform motion, the frequency of the reference signal is not constant. As mentioned above, in this case, the existing phase quadrature measurement method is not applicable. To solve this problem, a novel measurement method of nonlinearity based on a double-channel quadrature demodulation is presented, which is illustrated in Figure 2. This is realized in the field programmable gate array (FPGA). Compared with the traditional phase quadrature measurement method, there are two key distinctions. The first distinction is that the traditional method is based on a single lock-in amplifier while the new method adopted two lock-in amplifiers (a lock-in amplifier mainly consists of two mixers and two low pass filters). The second distinction is that, in the traditional method, the reference signal of the interferometer serves as the external reference of the lock-in amplifier while, in the new method, the external reference signal is abandoned. Instead, a pair of quadrature signals generated inside the FPGA are used as the reference signals of the lock-in amplifiers and both the reference and measurement signals of the interferometer serve as the measurement signals of the two amplifiers. 

The two quadrature signals generated inside the FPGA can be expressed as $I_{\cos}{\;=\;\cos\omega }_{0}t$ and $I_{\sin}{\;=\;\sin\omega }_{0}t$ where $\omega_{0}$ is the angular frequency. As shown in Figure 2, in the first step, the internally generated quadrature signals are mixed with reference and measurement signals of the heterodyne interferometer, respectively. This operation is performed by four mixers. After low pass filtering, the output of the mixers can be expressed by the equation below.

(3) $$\left\{ \begin{array}{l} \sin Q\;\;=\;\;-\;\;\frac{A}{2}\sin(\Delta \omega t-\Delta \varphi -\omega_{0}t)\;\;-\;\;\frac{\sum_{m}\alpha_{m}\;}{2}\sin\left(\Delta \omega t+\theta_{m}-\omega_{0}t\right)\\ \cos Q\;\;=\;\;\frac{A}{2}\cos(\Delta \omega t-\Delta \varphi -\;\;\omega_{0}t)+\frac{\sum_{m}\alpha_{m}}{2}\cos\left(\Delta \omega t+\theta_{m}-\omega_{0}t\right)\\ \sin H\;\;=\;\;-\;\;\frac{B}{2}\sin(\Delta \omega t+\Delta \varphi -\omega_{0}t)-\frac{\sum_{n}\beta_{n}}{2}\sin\left(\Delta \omega t+\delta_{n}-\omega_{0}t\right)\\ \cos H=\frac{B}{2}\cos(\Delta \omega t+\Delta \varphi -\omega_{0}t)+\frac{\sum_{n}\beta_{n}}{2}\cos\left(\Delta \omega t+\delta_{n}-\;\;\omega_{0}t\right). \end{array}\right.$$

Based on Equation (3), the cosine component *C*(*t*) and sine component *S*(*t*) can be calculated using the formula below.

(4) $$\left\{ \begin{array}{l} C(t)\;=\;\sin Q\sin H\;+\;\cos Q\cos H\\ \;\;\;\;\;\;\;\;\;=\;\frac{1}{4}AB\cos(2\Delta \varphi )\;+\;\frac{1}{4}A\sum_{n}\beta_{n}\;\cos(\Delta \varphi \;+\;\delta_{n})\\ \;\;\;\;\;\;\;\;\;\;\;\;\;\;+\;\frac{1}{4}B\sum_{m}\alpha_{m}\cos(\Delta \varphi \;-\;\theta_{n})\;+\;\frac{1}{4}\sum_{m}\alpha_{m}\sum_{n}\beta_{n}\cos(\delta_{n}\;-\;\theta_{m})\\ S(t)\;=\;\cos H\sin Q\;-\;\sin H\cos Q\\ \;\;\;\;\;\;\;\;=\;\frac{1}{4}\;AB\sin(2\Delta \varphi )\;+\;\frac{1}{4}A\sum_{n}\beta_{n}\sin(\Delta \varphi \;+\;\delta_{n})\\ \;\;\;\;\;\;\;\;\;\;\;\;\;\;+\;\frac{1}{4}B\sum_{m}\alpha_{m}\sin(\Delta \varphi \;-\;\theta_{m})\;+\;\;\frac{1}{4}\sum_{m}\alpha_{m}\sum_{n}\beta_{n}\sin(\delta_{n}\;-\;\theta_{m}), \end{array}\right.$$

then the amplitude and phase can be calculated using Equations (5) and (6).

(5) R(t)=C(t) 2+S(t)2=14{[ABcos(2Δφ)+A∑nβncos(Δφ +δn)+B∑mαmcos(Δφ − θm)+ ∑mαm∑nβncos(δn−θm)]2+[ABsin(2Δφ)+A∑nβnsin(Δφ + δn)+ B∑mαmsin(Δφ − θm) + ∑mαm∑nβnsin(δn−θm)]2}12,

and

(6) $$\begin{array}{l} \Phi (t)\;=\arctan\left[\frac{S(t)\;}{C(t)}\right]\\ \;\;\;\;\;\;\;=\arctan\frac{AB\sin(2\Delta \varphi )+A\sum_{n}\beta_{n}\sin(\Delta \varphi +\delta_{n})+B\sum_{m}\alpha_{m}\sin(\Delta \varphi -\theta_{m})+\sum_{m}\alpha_{m}\sum_{n}\beta_{n}\sin(\delta_{n}-\theta_{m})}{AB\cos(2\Delta \varphi )+A\sum_{n}\beta_{n}\cos(\Delta \varphi +\delta_{n})+B\sum_{m}\alpha_{m}\cos(\Delta \varphi -\theta_{m})+\sum_{m}\alpha_{m}\sum_{n}\beta_{n}\cos(\delta_{n}-\theta_{m})}. \end{array}$$

As the reference and measurement signals, *I_m_* and *I_r_* have equal but opposite DFS. The theoretical phase difference between them is 2Δφ. Therefore, the measurement error of phase is shown below.

(7) δφ(t)=Φ(t)−2Δφ.

By using the first order approximation of the Taylor expansion for Equation (6), Equation (7) is expressed by the formula below.

(8) $$\delta \varphi (t)\approx \frac{1}{A}\sum_{m}\alpha_{m}\;\sin(-\theta_{m}-\Delta \varphi )+\frac{1}{B}\sum_{n}\beta_{n}\sin(\delta_{n}-\Delta \varphi ).$$

Similarly, δ*R*(*t*)/*R*(*t*) can be expressed by the equation below.

(9) $$\frac{\delta R(t)\;}{R(t)}=\frac{R(t)-R_{0}}{R(t)}\;\;\;\approx \frac{1}{A}\sum_{m}\alpha_{m}\cos(-\theta_{m}-\Delta \varphi )+\frac{1}{B}\sum_{n}\beta_{n}\cos(\delta_{n}-\Delta \varphi ),$$

where $R_{0}$ is the amplitude when $\alpha_{m}{=\;\beta }_{n}=\;0\;(m,\;n=1,\;2,\;3,\;\ldots )$. Equations (8) and (9) are similar in mathematical expressions except for a 90° phase delay. Thus, in real applications, to evaluate the nonlinearity, we can calculate δ*R*(*t*)*/R*(*t*) rather than δφ(t) since the calculation of δ*R*(*t*)/*R*(*t*) are much easier to realize in practical applications. As shown by Equation (7), to calculate the phase error δφ(t), it is necessary to know the real phase 2Δφ. However, this is not easy to realize in practical applications because it is extremely difficult to provide a controlled displacement at nanometer or sub-nanometer level. Actually, in most of the practical applications, the real phase is an unknown value. However, for calculating δR(t)/R(t), it is not necessary to know the real phase. To evaluate the system nonlinearity, the phase delay is a negligible factor. Therefore, the nonlinearity can be calculated by the equation below.

(10) $$\delta L_{nonlin\;}=\;\frac{1}{M}\frac{\lambda }{2\pi }\delta \varphi (t)\;=\;\frac{1}{M}\frac{\lambda }{2\pi }\frac{\delta R(t)}{R(t)},$$

where *M* is the optical fold factor and λ is the laser wavelength for the heterodyne laser interferometers with bidirectional DFS, *M* = 4. For digital signals, δ*R*(*t*)/*R*(*t*) can be calculated by the formula below.

(11) $$\frac{\delta R(t)\;}{R\left(t\right)}=\frac{R(n)-\bar{R}}{\bar{R}},$$

where

(12) $$\bar{R}\;\;=\frac{1}{N}\sum_{n=1\;}^{N}R(n).$$

The above analysis shows the overall procedure of the proposed method for measuring the nonlinearity in heterodyne laser interferometers. By adopting internal references for the lock-in amplifiers, this method avoids the problems induced by frequency variation of external references, which means it is no longer limited by the motion state of the target. By utilizing double-channel quadrature demodulation, the method can be applied extensively to the new type heterodyne laser interferometers with bidirectional DFS. In addition, for phase demodulation, it is not a simple subtraction of the measured phases of the two lock-in amplifiers. Instead, the phase differential algorithm is employed to improve computing efficiency.

## 3. Experiment Validation

**Figure 3:**
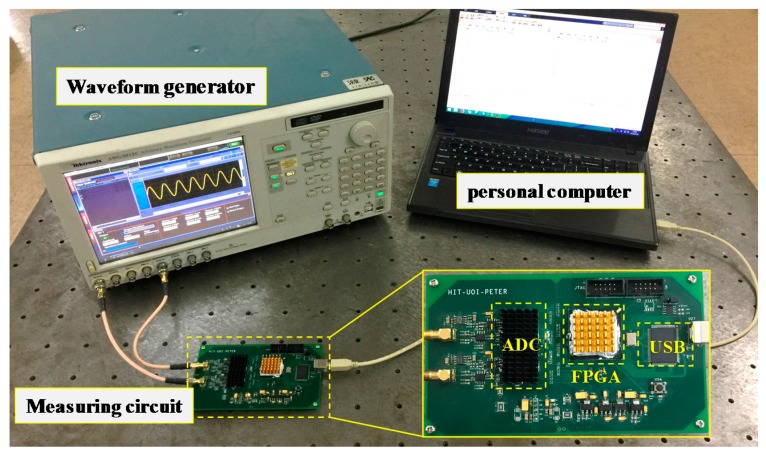
Experimental setup for validating the proposed measurement method of nonlinearity in heterodyne laser interferometers. ADC, analog-to-digital converter, FPGA, Field-Programmable Gate Array, USB, Universal Serial Bus.

To verify the proposed method, an experimental setup was established, which is shown in Figure 3, where the waveform generator (Tektronix, AWG5012C, Beaverton, OR, USA) is used to generate two signals that simulate the reference and measurement signals of the heterodyne laser interferometers. The measurement of the nonlinearity is performed by the measuring circuit in which the two signals from the waveform generator are first converted into digital signals by the analog-to-digital converters (ADC) (AD9446, Analog Devices, Norwood, MA, USA) and then are processed in the FPGA (EP3C120F780C8, Altera Corporation, Santa Clara, CA, USA). The method described in Section 2 is realized in the FPGA and the results are sent to the personal computer through a Universal Serial Bus (USB).

### 3.1. System Performance in Condition of Uniform Motion

To study the performance of the measurement system when the target is in uniform motion, the waveform generator produced two simulated signals, which are shown below.

(13) $$\left\{ \begin{array}{l} I_{r}=\underset{\mathrm{MRS}}{\underset{︸}{A\cos\left(2\pi \Delta ft-\Delta \varphi \;\right)}}+\underset{{\mathrm{PRS}}_{1}}{\underset{︸}{\alpha_{1}\cos\left(2\pi \Delta f-2\Delta \varphi \right)}}\;+\;\underset{{\mathrm{PRS}}_{2}}{\underset{︸}{\alpha_{2}\cos(2\pi \Delta ft-3\Delta \varphi )\;}}\\ I_{m}=\underset{\mathrm{MMS}}{\underset{︸}{B\cos\left(2\pi \Delta f+\Delta \varphi \right)}}+\underset{{\mathrm{PMS}}_{1}}{\underset{︸}{\beta_{1}\cos\left(2\pi \Delta f+2\Delta \varphi \right)}}+\underset{{\mathrm{PMS}}_{2}}{\underset{︸}{\beta_{2}\cos\left(2\pi \Delta f+3\Delta \varphi \right)}} \end{array}.\right.$$

In Equation (13), only the first-order and second-order nonlinear harmonics are retained because the higher order nonlinear harmonics are relatively quite small [12]. When the target moves at a constant velocity υ, the measured phase Δφ is calculated by the equation below.

(14) $$\Delta \varphi \;=\;2\pi \int f_{d}\;dt\;\;=\;\;2\pi \int \frac{2\upsilon }{\lambda }dt\;=\;\frac{4\pi \upsilon t}{\lambda },$$

where λ  = 632.8 nm.

**Figure 4:**
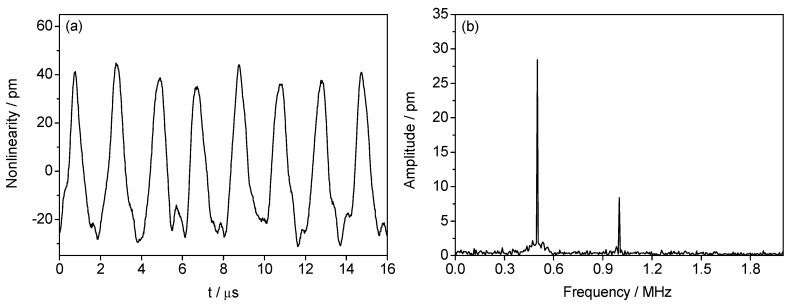
Experimental results of nonlinearities in condition of uniform motion with $∆{f=5\;\mathrm{MHz},\;f}_{d}=0.5\mathrm{MHz},\;A:\alpha_{1}{:\alpha }_{2}{\;=\;B:\beta }_{1}{:\beta }_{2}=\;10,000:6:2$. (**a**) Time domain and; (**b**) frequency domain.

In Equation (13), the reference signal $I_{r}$ consists of the main reference signal (MRS) and the parasitic reference signals (PRS_1–2_) and the frequency of MRS, PRS_1_, and PRS_2_ are $\Delta f-f_{d}$, $\Delta f-2f_{d}$, and $\Delta f-3f_{d}$, respectively. Similarly, the measurement signal $I_{m}$ consists of the main measurement signal (MMS) and the parasitic measurement signals (PMS_1–2_) and the frequency of MMS, PMS_1_, and PMS_2_ are $∆{f+f}_{d}$, $∆{f+2f}_{d},$ and $∆{f\;+\;3f}_{d}$, respectively. In this experiment, the beat frequency ∆f and the DFS $f_{d}$ are set as 5 MHz and 0.5 MHz, respectively. The equivalent velocity of the target is 0.1582 m/s. The ratio between the amplitudes of MRS, PRS_1_, and PRS_2_ is set as 10,000:6:2. For MMS, PMS_1_, and PMS_2_, the ratio of the amplitudes is also set as 10,000:6:2. By substituting these values into Equations (9) and (10), the theoretical magnitudes for the first-order and second-order nonlinearities are calculated as 30.2 pm and 10 pm, respectively. Figure 4a,b show the corresponding experimental results in time domain and frequency domain, respectively. The overall nonlinearity shown in Figure 4a is the superposition of the first-order and second-order periodic errors. By employing the Fast Fourier Transform (FFT) to the data in Figure 4a, the frequencies and amplitudes for the first-order and second-order nonlinearities can be determined. As shown in Figure 4b, the frequency and amplitude for the first-order nonlinearity are 0.5 MHz and 28.42 pm, respectively. For the second-order nonlinearity, the frequency and amplitude are 1.0 MHz and 8.42 pm, respectively. Therefore, in this case, the measurement errors for the first-order and second-order nonlinearities are 1.78 pm and 1.58 pm, respectively.

### 3.2. System Performance in Condition of Non-Uniform Motion

To study the performance of the measurement system when the target is in non-uniform motion, the waveform generator produced two simulated signals identical to that in Equation (13). When the target moves with a constant acceleration *a*, the measured phase Δφ is calculated by the equation below.

(15) $$\Delta \varphi \;=\;2\pi \int f_{d}\;dt\;=\;2\pi \int \frac{2at}{\lambda }dt\;=\;\frac{2\pi at^{2}}{\lambda }.$$

**Figure 5:**
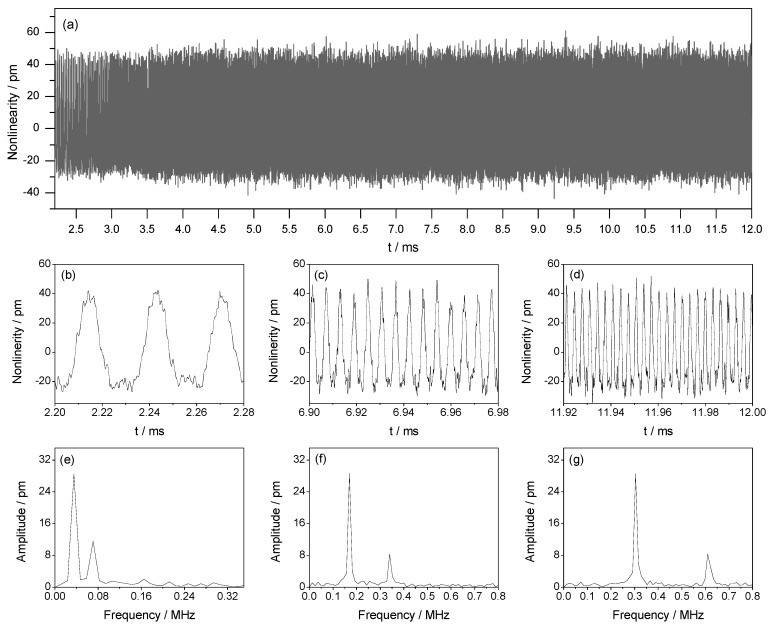
Experimental results of nonlinearities in condition of non-uniform motion with $∆f=5\;\mathrm{MHz},\;a=5\;{\mathrm{m/s}}^{2}{,\;A:\alpha }_{1}{:\alpha }_{2}{\;=\;B:\beta }_{1}{:\beta }_{2}=\;10,000:6:2$. (**a**) time domain; (**b**–**d**) partial enlarged drawings for the beginning, middle, and end parts of (**a**) with time length of 0.08 ms; (**e**–**g**) spectrums of (**b**–**d**), respectively.

In this experiment, the beat frequency ∆f is set as 5 MHz and the target acceleration is set as 5 m/s^2^. In the reference signal *I_r_*, the ratio between the amplitudes of MRS, PRS_1_, and PRS_2_ is set as 10,000:6:2. In the measurement signal *I_m_*, the ratio between the amplitudes of MMS, PMS_1_, and PMS_2_ is also set as 10,000:6:2. With these given values, the theoretical magnitudes of the first-order and second-order nonlinearities can be calculated as 30.2 pm and 10 pm, respectively. Figure 5a shows the measured nonlinearities in the time domain with the sampling times of 9.8 ms. Figure 5b–d is the partial enlarged drawings of the beginning, middle, and end parts of Figure 5a. The time length of each part is 0.08 ms. It can be seen that, with the increase of time, the period of the measured nonlinearity decreases, which indicates an accelerated motion of the target. Since the time length in Figure 5b–d is very short, the target velocity in each panel can be considered constant. By applying the FFT to the data in Figure 5b–d, the first-order and second-order nonlinearities can obtained, which are presented in Figure 5e–g, respectively. The frequencies of the first-order nonlinearity in Figure 5e–g are 0.035 MHz, 0.170 MHz, and 0.305 MHz, respectively, and the corresponding amplitudes are 28.50 pm, 28.61 pm, and 28.57 pm, respectively. Similarly, the frequencies of the second-order nonlinearity in Figure 5e–g are 0.070 MHz, 0.340 MHz, and 0.610 MHz, respectively, and the corresponding amplitudes are 11.61 pm, 8.35 pm, and 8.39 pm, respectively. Both the first-order and second-order nonlinearities calculated from the above-mentioned three parts are close to the theoretical values and the max measurement error is about 1.7 pm, which indicates a good reliability of the proposed method.

## 4. Conclusions

Measurement method of nonlinearity plays an essential role in the development of the heterodyne laser interferometers with ultra-high accuracy. The application of the existing methods is restricted by the type of interferometers and the motion state of the target. To break through these limits, a novel measurement method is proposed in this study. By employing the double-channel quadrature demodulation together with internal reference signals, this method is free from the heterodyne laser interferometer and the motion state of the target. Additionally, for phase demodulation, the phase differential algorithm is utilized to improve the computing efficiency. The experimental results show that the proposed measurement method achieves accuracy better than 2 pm. This method is expected to benefit the development of the next-generation laser interferometers.
